# Approaches to Peripheral Nerve Repair: Generations of Biomaterial Conduits Yielding to Replacing Autologous Nerve Grafts in Craniomaxillofacial Surgery

**DOI:** 10.1155/2016/3856262

**Published:** 2016-07-31

**Authors:** Robert Gaudin, Christian Knipfer, Anders Henningsen, Ralf Smeets, Max Heiland, Tessa Hadlock

**Affiliations:** ^1^Division of Facial Plastic and Reconstructive Surgery, Department of Otolaryngology/Head and Neck Surgery, Harvard Medical School, Massachusetts Eye and Ear Infirmary, 243 Charles Street, Boston, MA 02114, USA; ^2^Department of Oral and Maxillofacial Surgery, University Medical Center Hamburg-Eppendorf, Martinistrasse 52, 20246 Hamburg, Germany; ^3^Department of Oral and Maxillofacial Surgery, Bundeswehrkrankenhaus Hamburg, Lesserstraße 180, 22049 Hamburg, Germany

## Abstract

Peripheral nerve injury is a common clinical entity, which may arise due to traumatic, tumorous, or even iatrogenic injury in craniomaxillofacial surgery. Despite advances in biomaterials and techniques over the past several decades, reconstruction of nerve gaps remains a challenge. Autografts are the gold standard for nerve reconstruction. Using autografts, there is donor site morbidity, subsequent sensory deficit, and potential for neuroma development and infection. Moreover, the need for a second surgical site and limited availability of donor nerves remain a challenge. Thus, increasing efforts have been directed to develop artificial nerve guidance conduits (ANCs) as new methods to replace autografts in the future. Various synthetic conduit materials have been tested* in vitro *and* in vivo,* and several first- and second-generation conduits are FDA approved and available for purchase, while third-generation conduits still remain in experimental stages. This paper reviews the current treatment options, summarizes the published literature, and assesses future prospects for the repair of peripheral nerve injury in craniomaxillofacial surgery with a particular focus on facial nerve regeneration.

## 1. Introduction

This educational paper provides an overview of the evolvement of current approaches for the rehabilitation of nerve defects by means of artificial nerve guidance conduits (ANCs) and gives an outlook on their clinical application in craniomaxillofacial surgery with special regard to the facial nerve.

**Figure 1:**
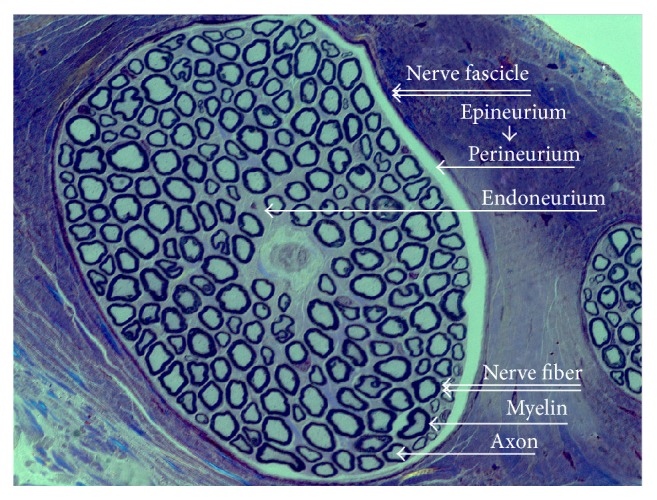
Cross section buccal branch of the facial nerve, Toluidine Blue, 40x/1.3 oil, Zeiss Microscope.

Peripheral nerve injury is caused by a myriad of conditions including trauma, tumor, and iatrogenic injury. Over 200,000 peripheral nerve repairs are performed annually in the USA [1]. In craniomaxillofacial or facial plastic surgery, damage to peripheral nerves frequently involves the facial nerve. These injuries result from thermal, ischemic, mechanical, or chemical damage (to the nerves). Viral infections such as simple herpes and herpes zoster, trauma, inflammatory infections of the middle ear, metabolic diseases, and tumors can lead to nerve defects. In the facial area peripheral facial paralysis (PFP) resulting from affection of the seventh nerve is the most common pathology of the cranial nerves. Its incidence ranges from 20 to 30 cases per 100,000 people. Regeneration of the peripheral nervous system (PNS) after injury has a much better outcome compared to the central nervous system (CNS). Whereas CNS injury results in a glial scar that restrains axonal growth, following PNS injury, a more optimal environment for axonal outgrowth exists [2]. Surgeons most often use the Sunderland classification to categorize nerve injury when developing an appropriate treatment plan [3]. The Sunderland classification includes five grades of nerve injury (Table 1, Figure 1). Sunderland 1 and 2 injuries result in complete recovery, whereas in grades 3 to 5 Wallerian degeneration takes place, which is followed by aberrant regeneration of varying degrees. Wallerian degeneration, which is initiated immediately after injury, consists of myelin sheath degradation. Following injury, severed axon ends are sealed and the regenerative phase is initiated [4]. Within a few hours, the proximal portion of the severed nerve initiates a regenerative response with axonal outgrowth that migrates to the distal portion, which degenerates after the latent phase of the injury. However, concerning the specific regeneration pathway that leads to a correct matching of axon and end organ, Witzel et al. reported that even after direct end-to-end suturing of the lesioned mouse sciatic nerve, only 17% of the regenerating axons had crossed the repair site by 5 days [5]. Whether there is a linkage between the Wallerian degeneration (after axonal transection) and the large group of peripheral nerve diseases known as “dying back” neuropathies, in which axon degeneration is also most prominent in distal nerves and spreads in a retrograde manner, is unclear [6]. The question remains whether all axonal degeneration processes, no matter if this is through transection or toxic and genetic disorders, follow the same cascade of changes as seen in Wallerian degeneration [7–9]. Due to the denervation of adjacent muscles and tissues, there is a loss of motor function and sensation to the previously innervated area [10]. These patients have a decreased quality of life stemming from neuropathies and, in the case of facial nerve injury, acquired conditions known as flaccid and/or nonflaccid facial paralysis, synkinesis (Table 2), or chronic pain. Sunderland 3 injury is mostly often treated medically, whereas Sunderland 4 and 5 injuries are usually treated surgically, with neurolysis and reconstruction of the defect [11]. Extensive research has resulted in new strategies, which have improved prognosis and encouraged the natural nerve regeneration process. The current gold standard of treatment is transplantation of a nerve autograft to bridge the defect. However, availability of appropriate donor nerves is limited and requires an additional surgical site. Associated donor site morbidity, subsequent sensory deficit, neuroma development, and infection risk thus impair the applicability of autografts [12, 13]. Additionally, only 40–50% of the patients treated with autologous nerve grafts regain an acceptable level of function [14]. Therefore, research focuses on designing nerve conduits that act as splints, encouraging and fastening regrowth of the transected nerve and additionally forming a barrier to ingrowth of connective tissue [15]. This educational paper intends to clarify and outline the most important aspects of artificial nerve guide conduits, explain their historical relevance, and discuss optional prospects in craniomaxillofacial surgery.

## 2. Classical Approaches to Address Peripheral Nerve Injuries

Important parameters for the regeneration of peripheral nerves after injury include the width of the nerve gap, the length of elapsed time from injury to treatment, and the patient's age and comorbidities affecting the nervous and circulatory systems. If the nerve gap is less than 5 mm, spontaneous regeneration of axons is possible [16]. Direct end-to-end coaptation (neurorrhaphy) can be applied only in small gaps since tension across the suture lines is known to inhibit regeneration [17, 18]. However, a decision has to be made whether a direct epineurial and/or group fascicular end-to-end repair via suturing is possible, to offer a peripheral nerve autograft or bridge the defect with a nerve conduit to guide axonal regrowth [19–22]. For facial reanimation a variety of nerve autografts have been used throughout the last century. The techniques advanced from unilateral to a contralateral cross facial nerve graft [23]. In 1879, Drobnick performed the first reported successful unilateral nerve graft using the spinal accessory nerve [24]. After a few decades the accessory-facial nerve crossover was replaced by the hypoglossal-facial nerve crossover as described by Körte and Bernhardt in 1903 [25]. In 1924, Ballance harvested the recurrent laryngeal nerve for a crossover with the facial nerve [26]. In 1996, Scaramella utilized the contralateral facial nerve to drive the paralyzed facial nerve [27]. They introduced the cross facial nerve graft using the sural nerve as an autograft, which has since then been the gold standard for facial reanimation in facial plastic surgery. The procedure also has a sufficient success rate in regeneration of larger nerve gaps [28–30]. If the sural nerve has already been harvested or additional grafting material is required, the saphenous nerve, the great auricular nerve, or the medial antebrachial cutaneous nerve is possible alternatives [31].

## 3. Nerve Grafts

Bridging larger gaps is possible using autografts, allografts, and xenografts. Autografts offer the best opportunity for nerve reconstruction [43]. Allografts and xenografts may be reconstructive options but require sufficient immunosuppression [44]. These options bear risks of cross contamination, secondary infection, or immune rejection and special processing for decellularization is needed [30, 45–47]. As a result of immunosuppression, patients may be more susceptible to infections and formation of secondary malignancies.

Sensory nerves that have minor roles and provide excellent guidance features for axonal regrowth, such as the sural nerve, are harvested for use as autografts [48]. Frerichs et al. and Kim at al. have shown that acellular allogenic nerve grafts are very effective for gaps of 1-2 cm in a rat sciatic model [49, 50]. One such Food and Drug Administration (FDA) approved allogenic nerve graft is Avance®, which is produced by AxoGen, Inc. (Alachua, FL, USA) (Table 3). In another* in vivo*-study Avance was superior to a currently available second-generation nerve conduit but failed to confer the regenerative advantages of an isograft [51]. AxoGuard*™* is the only porcine small intestine submucosa (SIS) extracellular matrix coaptation aid with FDA-approval. In a preliminary study using a rat sciatic model, distally directed growth of the proximal nerve was determined histologically [52]. In a further* in vivo*-study, results of SIS were histologically superior to silicone tubes and the SIS-group showed better EMG-response for distal motor latency and amplitude than the silicone group [53]. However, controlled clinical trials are still needed to determine the success of both treatment options in comparison to nerve conduits and nerve allografts.

## 4. Nerve Conduits as Approaches to Repair Peripheral Nerve Injuries

**Figure 2:**
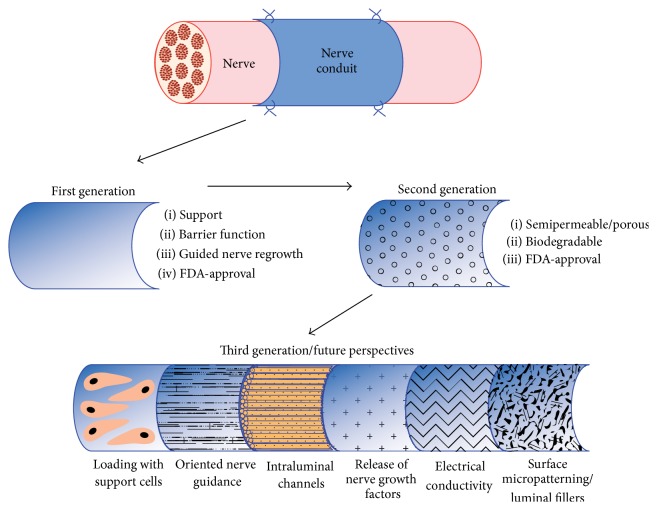
Generations of nerve conduits and future perspectives.

Due to the disadvantages of autografts, allografts, and xenografts and with progress in regenerative medicine and tissue engineering, various artificial and biological nerve conduits have been developed. Brunelli et al. defined four factors for an ideal nerve conduit material: (1) compatibility to the surrounding tissues, (2) easy preparation to fitted length and size, (3) incorporated chemotactic substances for nerve outgrowth attraction as well as giving a basis for axonal regeneration, and (4) protection against scar tissue infiltration [54]. According to Arslantunali et al. an ideal nerve conduit should have properties like flexibility, biocompatibility, biodegradability, high porosity, neuroinductivity, neuroconductivity, easy handling, and sufficient endurance [45]. Autologous nonnerve tissue, such as bone, artery, vein, or muscle, has been successfully used as a viable option for nerve conduits. Glück was the first to provide a channel across a nerve gap by decalcified bone in 1880 [55]. Bünger reported a successful use of the brachial artery as a conduit for a sciatic nerve defect in dogs in 1891 [56]. Wrede was the first to report the use of a 45 mm vein conduit in 1909 [57]. The first use of a skeletal muscle autograft for bridging of nerve gaps was reported in 1940 [58]. However, several disadvantages such as low outcome, lack of suitable donor vessels, venous lumen collapse, growth of nerve fibers out of the muscle tissue during regeneration, and the necessity of a donor site have shifted the focus of research onto biological and artificial nerve conduits [59].  Figure 2 gives a general overview of the different nerve conduits generations. Experimental and clinical results in a great number of studies have shown that the clinically available nerve conduits can induce comparable or even superior nerve reconstruction results as compared to nerve autografts when nerve defect gaps are small [43]. Yet, a satisfactory outcome in bridging larger nerve defects still remains a challenge. The critical defect gap size for a conduit to have sufficient nerve regeneration and functional recovery is between 1.5 and 3 cm [1, 13, 30, 54]. Data obtained from different research groups are difficult to compare in the absence of any widely accepted standardization of animal models and investigative methods. Thus, even if multiple studies demonstrating sufficient regeneration with the use of conduits in bridging nerve gaps exist, limitations with regard to their critical defect size and diameter as well as differences in the regenerative potential of different animal models as well as interindividual differences in the study approach and methods of the different workgroups have to be taken into account when interpreting the results. Nectow et al. provide a comprehensive review of the critical defect size and its role in nerve regeneration through nerve conduits [60]. The significant regenerative challenge with implanted devices promoting axonal regeneration in critical size defects led to the development of new, alternative designs. These nerve conduit models mimic the features of a natural nerve, with additional growth/neurotrophic factors, cells, nucleic acids, and ECM molecules such as collagen, laminin, and fibronectin (Figure 2).

## 5. First-Generation Nerve Conduits

The initial strategy to develop a synthetic conduit was to design a support structure that would guide regrowth of the transected nerve and provide a stable barrier against the infiltration of connective tissue [61, 62]. The first artificial conduit generation was tubes of nonresorbable silicone or polytetrafluoroethylene (ePTFE, Gore-Tex®) [21]. S. Stanec and Z. Stanec found that ePTFE conduits are a reliable and successful surgical treatment option for nerve gaps up to 4 cm in humans [63]. Lundborg et al. reported successful ulnar nerve repairs which involved very short nerve gaps (2-3 mm) with silicone tubes but often required secondary removal surgeries due to compression syndrome or fibrotic encapsulation of the implant [64–66]. Newer approaches are fillings of synthetic conduits with structural proteins, blood components, stem cells, or messenger substances, but knowledge is still limited and further studies are needed to evaluate the advantages of these modifications [67–69].

## 6. Second-Generation Nerve Conduits

Second-generation nerve conduits are constructed from resorbable material, are biocompatible, and have specific tube wall structures. These conduits aim to increase functional rehabilitation and axonal remyelination through enhancement of material biocompatibility and topography. Subsequent research focused mainly on identifying various resorbable materials to avoid a second-stage surgery. The second generation of artificial nerve conduits therefore is hollow tubes consisting of different biocompatible materials such as resorbable type I collagen, polyglycolic acid (PGA), and poly-DL-lactide-co-caprolactone (PLCL) as well as nonresorbable polyvinyl alcohol (PVA).

### 6.1. Polyglycolic Acid

The polyglycolic acid (PGA) conduit was the first clinically available bioabsorbable conduit (NeuroTube; Synovis Micro Companies Alliance, Birmingham, Ala.) (Table 3). The material is more flexible than silicone and the porosity allows oxygen diffusion, which is critical to the regeneration process. The degradation time is approximately six to twelve months [70]. However, when this type of reconstruction was performed in humans, the first conduits were utilized only to reconstruct sensory nerve defects. Dellon and Mackinnon demonstrated positive results after 1 year in a 3 cm ulnar nerve gap in monkeys using a second-generation nerve conduit compared to autografts [71]. Weber et al. found in a randomized prospective multicenter evaluation of digital nerve repair that PGA conduits were equivalent or superior to traditional autografts for reconstruction of long sensory nerve gaps up to 3 cm [72]. For short gaps less than 4 mm PGA conduits achieved superior results when compared to end-to-end repair. Rosson et al. demonstrated that bioabsorbable PGA nerve conduits can be offered to regenerate small motor nerve defects successfully [73]. Several case reports confirm good results in reconstruction of peripheral nerve defects up to 3 cm with PGA conduits [74–76]. However, there is a concern that PGA conduits might degrade before the nerve regeneration process is completed and that its lactic acid degradation product may have toxic effects [59, 77]. Extrusion of PGA conduits has also been reported [78].

### 6.2. Type I Collagen

Collagen is the most commonly used material for conduit fabrication. It is an important structural protein that is found ubiquitously in the body, for example, as fibrils in the endoneurium or as a nonfibrillar component in the basal lamina [79]. It supports tissue healing and cellular proliferation [21]. Collagen hydrogels are generated from fibrillar collagen sheets, which are rolled into three-dimensional nerve conduits [45, 80, 81]. Collagen conduits are capable of splinting small nerve defects up to 20 mm [82–85]. In a significant number of* in vivo*-studies using rat, cat, dog, and primate models, collagen conduits showed good functional outcomes in nerve reconstructions [86–89]. Tyner and colleagues found that collagen nerve conduits may reduce the severity of symptoms associated with neuropathic pain and alter the regrowth of transected nerves [90]. Favorable outcomes have also been reported in several clinical studies [82–85]. Taras et al. showed a good nerve recovery with the use of collagen conduits in 73% of patients, bridging a 5–15 mm digital nerve gap [91]. In a small case series the outcome of reconstructions with collagen conduits in infants suffering from plexus brachialis injury during birth was analyzed [92]. Four of the five patients experienced a good motor recovery after 1 year and three of the five patients had excellent recovery after 2 years of follow-up. Currently there are five commercially available FDA approved collagen type one nerve conduits: NeuraGen, NeuroMatrix, NeuroFlex, NeuraWrap, and NeuroMend (Table 3). Some collagen conduits have a degradation time as long as 48 months, while most have a short degradation time of four to eight months. This raises the concern that degradation may occur before nerve regeneration process is completed depending on the size of the nerve defect [93]. Better results were obtained in long-term recovery of sensation after digital nerve reconstruction and use of a collagen conduit in short gaps below 10 mm when compared to long gaps [94].

### 6.3. Caprolactone Conduits

A poly-D,L-lactide-co-epsilon-caprolactone conduit (P(LL-co-CL)) consists of lactic acid and caprolactone monomers. Neurolac® is the only currently FDA approved synthetic caprolactone conduit (Table 3). These transparent conduits produce fewer toxic degradation side products and have a long degradation time up to 16 months [95]. In a rat model, only very small fragments could be found 16 months after reconstruction of the sciatic nerve, but neither fragments nor foreign body reaction appeared to influence the regeneration process [96]. However, Duda and colleagues in 2014 reported a strong foreign body response that interferes with peripheral nerve regeneration [97]. The efficacy of P(LL-co-CL) nerve guides has been assessed with mixed outcomes. den Dunnen et al. and Meek et al. demonstrated favorable results in nerve reconstruction in the sciatic rat model [98–103]. Shin et al. bridged a 10 mm sciatic nerve defect in 80 Levis rats comparing three established synthetic conduits P(LL-co-CL) conduit, type I collagen conduit, and a PGA conduit with a reversed autograft and reported similar outcomes for P(LL-co-CL) conduits compared to the autografts. Chiriac and colleagues showed a poor recovery rate in 28 human upper extremity nerve repairs with P(LL-co-CL) conduits [104, 105]. However, it has to be kept in mind that one main factor thought to be associated with outcome after the repair of peripheral nerve injuries is the location of the injury. An injury close to the proximal end of the nerve has a poor functional recovery after nerve repair, whereas an injury close to the distal end has a good recovery rate. Secer et al. conducted a study on 455 patients with ulnar nerve injuries and good to excellent results were achieved in 15.06% of high-level injuries, 29.60% of mid-level injuries, and 49.68% of low-level injuries [106]. An explanation for this is that mixed nerve bundles are often found in the proximal segment of the nerve tract, which then divide into sensory and motor tracts at the distal end. Therefore, risk of crossover growth between sensory and motor nerve fibers is high which becomes a greater challenge in restoring function following a proximal lesion. Additionally, more proximal injuries take a longer time to regenerate due to a greater distance to the nerve ending. This leads to increased difficulty in restoring sensory or motor function after an extended period of denervation [107].

Compared to single P(LL-co-CL) conduits, the filling of P(LL-co-CL) conduits with muscle tissue in a 10 mm sciatic nerve rat model (*n* = 25) in a study conducted by Varejão et al. showed no superiority. Meek and colleagues reported superior results of the modified conduits in an investigation of a 15 mm sciatic rat model (*n* = 30) [108–111]. Furthermore, caprolactone conduits with varying degrees of porosity showed no difference in outcome based on the porosity [112]. The efficacy of P(LL-co-CL) conduits is therefore controversial and controlled clinical trials are needed.

### 6.4. Polyvinyl Alcohol (PVA)

PVA conduits have been approved by the US Food and Drug Administration (FDA) as the only nondegradable synthetic nerve guide (SaluBridge; SaluTunnel; SaluMedica LLC, Atlanta, GA, USA) (Table 3). So far, no information about the repair efficacy of these conduits has been reported in peer-reviewed journals [45, 60, 113].

### 6.5. N-Fibroin

Silk protein, N-fibroin, is produced initially as a soluble protein in the glands of silk worms and later arranged into fibrous structures during the spinning process. N-fibroin has been rigorously studied with superior biocompatibility and low immunogenicity, and it is degradable with excellent mechanical stability [114]. For nerve repair it must be integrated in a tubular-like structure to provide a guidance channel for sprouting axons and to protect nerve fibers and their neurotrophic factors from infiltration of fibrous tissue [115]. Several recent studies demonstrated a good potential for N-fibroin and other silk proteins in reconstruction of peripheral nerves* in vitro* and* in vivo* [116–118]. Yang and colleagues developed and tested a silk fibroin based nerve guidance conduit with oriented filaments which yielded successful outcomes in the rat sciatic model [119]. Huang et al. showed similar functional and histological results of silk conduits when compared to nerve autografts* in vivo* [114]. Mottaghitalab et al. fabricated an oriented tubular substitute by means of freeze-dried silk/single walled carbon nanotubes and reconstructed a 10 mm nerve gap in the rat sciatic model [120]. In a recent study, Das and colleagues showed that a newly developed, silk-based gold nanocomposite conduit preseeded with Schwann cells performed well in terms of structural and functional regeneration of severed sciatic nerves* in vivo* [121]. This finding is in accordance with earlier studies which show enhanced cell-material interactions like cell adhesion, proliferation, and differentiation by chemically modified silk nanofibers with gold nanoparticles [122]. Gold nanoparticles are thought to immobilize specific molecules of the nanofiber without generating significant cytotoxicity. Furthermore, an enhancement in cellular adhesion and spreading on the modified surface can be attained [122]. Animals were even able to perform complex locomotor activities with an outstanding sciatic function index. Subsequently, several recent studies have been published in which superior results of silk fibroin conduits loaded with nerve growth factors were demonstrated [123–128]. Despite the very promising results of N-fibroin conduits neither the FDA nor any other administration has approved any silk conduit.

## 7. Third-Generation Nerve Conduits

Although second-generation nerve conduits provide sufficient guidance for the regeneration of nerve defects, there are mixed results on their efficacy when compared to autologous nerve grafts. Second-generation nerve conduits are simple hollow tubes, which do not possess the characteristics of an autograft [14]. The third generation of conduits, which is not FDA approved and is the present focus of research, represents artificial conduits that may incorporate controlled release/delivery of neurotrophic factors, electroconductive material, stem or Schwann cells, extracellular matrix proteins, surface micropatterning, or luminal fillers as guidance structures with favorable physical and mechanical properties [129].

## 8. Surface Micropatterning and Extracellular Matrix Proteins

Surface micropatterning and the inclusion of extracellular matrix proteins are new techniques to provide the most suitable nanostructure topography for adequate neural growth and to simulate topographical dimensions similar to the nerve extracellular matrix [129, 130].

Electrospinning is a frequently used technique to fabricate matrices with imprinted micropatterns. This technique yields a greater area-to-volume ratio in the conduit compared to smooth surface scaffolds. The greater area-to-volume ratio leads to significantly greater adsorption of adhesion molecules, leading to enhanced cell attachment (e.g., Schwann cells) [131–133]. Furthermore, by controlling the architecture of the pore size in the nerve scaffold wall, it is possible to develop a microporous inner layer and macroporous outer layer, resulting in bidirectional permeability [129]. Another advance in tissue engineering of scaffolds is the combination of extracellular matrix proteins (e.g., fibronectin, laminin, and collagen) with biodegradable polymers. This gives the normally hydrophobic scaffold a hydrophilic surface which is advantageous for controlled cell signaling [134]. Due to infection risk and immune rejection, peptides have been developed that mimic the active binding domains of various extracellular matrix molecules [135, 136]. For example, the arginine-glycine-aspartic acid (RGD) cell adhesion sequence is a fibronectin cell attachment site peptide and, like other such peptides, promotes the secretion of neurotrophic factors and cell attachment [137–139].

### 8.1. Luminal Fillers

Luminal fillers are growth guidance scaffolds in the lumen of the conduit that have been shown to be favorable in nerve repair [62, 140]. Neal et al. used electrospun blended PCL-laminin fiber nanomeshes with a diameter of 100 to 200 nm to repair 10 mm nerve gaps in the tibial nerve in a rat model [134]. Recovery with aligned nanofibers was superior to that with randomly aligned fibers. Furthermore, Arai et al. showed that aligned luminal fibers have a positive effect on nerve regeneration through stabilization of the matrix [141]. Another recent advance has been to add multiple longitudinal microchannels within the nerve conduit reducing axonal “dispersion” [129, 142]. Sundback et al. introduced a method to produce a conduit composed of a high-molecular-weight copolymer of lactic and glycolic acids (PLGA) with 100 longitudinally aligned channels by using a combined injection molding thermally induced phase transition technique [143]. These macropores are up to 20 *μ*m wide and provide a larger surface area and were better at supporting Schwann cell adherence compared to a single channel conduit [143]. Hadlock et al. designed a novel PLGA conduit with either 1, 5, 16, or 45 longitudinally aligned channels through a foam processing technique, using low-pressure injection molding. Additionally, Schwann cells were seeded into the conduit. This conduit was tested in a 7 mm sciatic nerve gap rat model and showed favorable outcomes in regeneration compared with autografts [144]. Bozkurt et al. introduced a novel nerve guide (Perimaix, Matricel GmbH, Herzogenrath, Germany), which is prepared by “unidirectional freezing” of porcine collagen harvested from animals. Via directed ice crystal growth through the collagen material, longitudinal micropores are created and the pore size can be adjusted between 20 *μ*m and 100 *μ*m. Better alignment of Schwann cells within the microchannels and good nerve regeneration results were observed 6 weeks after implantation [14].

In addition, the nerve regeneration process is also influenced by the stiffness of the scaffolds. Studies have shown that neural cells do not extend well on stiff membranes due to increased mechanical traction. Current literature suggests a range of 5 Pa to 50 Pa where neural cells extend well, but further studies are needed for clarification of the underlying mechanisms and specification of the parameters influencing neural cell extension [129, 145–147].

### 8.2. Stem Cells/Schwann Cells

Stem cell therapies have received increased attention in regenerative medicine [148–162]. Novel studies and techniques in stem cell biology enabled the reprogramming of somatic cells (e.g., skin fibroblasts) into induced pluripotent stem cells (iPSCs) [163]. iPSCs are useful for patient-specific cell therapies as they possess unlimited expansion potential. Thus, it is possible to derive expandable multipotent stem cells such as neural crest stem cells, which in turn can be differentiated into Schwann cells to facilitate the myelination of axons and to promote nerve regeneration. Furthermore, combining iPSCs and engineered scaffolds may result in a superior therapeutic effect and shows valuable potential for regenerative medicine and tissue engineering [164].

Stem cells promote various processes, such as wound healing and neuroregeneration [95, 165–167]. Schwann cells seeded in nerve conduits have been successfully used for nerve reconstruction [144, 168, 169]. However, the source is limited and a secondary surgery is required. Stem cells are able to secrete neurotrophic factors and provide a favorable microenvironment for neurogenesis and the proliferation of Schwann cells in peripheral nerve repair [170]. Stem cells can even be differentiated to have a Schwann cell-like phenotype [171]. Several animal model studies have suggested the advantage of stem cells in reconstruction of peripheral nerves. Shi et al. demonstrated significantly better functional outcome bridging a facial nerve gap in rats with a biodegradable PGA conduit filled with neural stem cells overexpressing glia-derived neurotrophic factor, compared with empty conduits [172]. PGA tubes filled with Schwann-like cells or bone marrow stem cells both had superior effects in regeneration of facial nerves in rats compared with PGA tubes alone [173]. PLGA tubes containing dental pulp stem cells were superior to PLGA tubes alone [174]. Similar effects could be found in rat sciatic models using differentiated adipose-derived stem cells in collagen conduits [175, 176]. Transplantation of stem cells* in vivo* therefore has potential as a successful adjunctive therapy [168]. PGA, PLCL, and collagen are the FDA approved materials most frequently used for culturing stems cells in nerve conduits. In addition to the requirements that would be necessary to obtain FDA-approval, no standards have been developed to date regarding which stem cell populations, treated with which techniques, could be added to nerve conduits to predictably improve results as compared to nerve conduits without stem cell treatment.

### 8.3. Neurotrophic Factors

There is a complex milieu of growth factors and cytokines in the regulatory process of tissue regeneration. Therefore, the use of nerve growth factors is of great clinical interest [15]. Numerous studies have been done on delivery systems including topical administration, subcutaneous injection, microosmotic pump, and diffusion or affinity-based polymer microspheres [177–185]. A successful approach in the engineering of an effective conduit has been accomplished by loading neurotrophic substances into the lumen or into the wall of the conduit. Nerve growth is promoted through controlled release of the neurotrophic factors via diffusion into the lumen directly to the site of injury [186].

To date, there have been multiple neurotrophic factors identified including transforming growth factor beta superfamily (TGF-*β*), nerve growth factor (NGF), neurotrophins 3, 4, and 5 (NT-3/4/5), ciliary neurotrophic factor (CNTF), neuregulin-1 (NRG1), brain-derived neurotrophic factor (BDNF), and glial cell line-derived neurotrophic factor (GDNF). These neurotrophic factors play essential roles in the development and regeneration of neurons [187].

### 8.4. NGF

Braun et al. found no effect of NGF on motor neurons in an* in vitro* study coculturing human skeletal muscle myotubes and rat embryo spinal cord [188]. NGF promotes neurite outgrowth of cultured motoneurons only in the presence of astrocytes [189]. On the contrary Lee et al. showed that when used with a delivery system NGF enhanced axonal regeneration of the sciatic nerve of rats [182, 190].

### 8.5. BDNF

BDNF has neuroprotective effects and was able to prevent the death of axotomized motor neurons in newborn rats* in vivo *[191]. Novikov et al. demonstrated that BDNF enhanced the regeneration of rat spinal motor neurons [192]. Therefore, endogenous BDNF acts as a survival factor for injured motoneurons [189, 193]. Even with a single exogenous dose of BDNF at the time of the injury a long-term protective effect on adult motor neuron survival may be possible* in vivo* [194]. Contrarily, a local continuous long-term low dose application of BDNF had no effect on the number of regenerating motor neurons. In literature the dose dependency of exogenous BDNF application has been studied. High dose BDNF application (12 ± 20 mg/day for 28 days) has been observed to interact with p75 receptors that serve to inhibit axonal regeneration. Subsequently high dosage of BDNF is said to promote inhibitory effects on axonal regeneration [195]. In a study by Moir et al., exogenous BDNF application after two weeks of delayed peripheral nerve regeneration showed a greater axonal diameter compared to application after immediate repair but did not show significant difference in functional outcome [196]. Anti-BDNF antibody also is observed to significantly reduce the length of regenerating axons, the number and density of myelinated axons, and the amount of sensory axon regeneration [197].

### 8.6. GDNF

GDNF rescues and prevents atrophy of axotomized facial motor neurons and is believed to be a potent protective factor against axotomy-induced motor and sensory neuron death [15, 198]. Overexpression of GDNF in developing muscle increases the number of motor axons innervating neuromuscular junctions* in vivo *[14, 199–206]. Oppenheim et al. demonstrated that GDNF can rescue developing avian motor neurons from natural programmed cell death* in vivo *[207]. Subcutaneous injection of GDNF during first postnatal weeks increases motor axon branching and muscle hyperinnervation [208]. The adjunctive use of GDNF after injury of the facial nerve in rats showed superior survival of motor neurons, a greater number of myelinated axons, and a better functional outcome compared to standard treatment [209].

### 8.7. CNTF

CNTF is also capable of prolonging survival and improving muscle function after nerve injury [210]. Atrophy and tetany of denervated muscle are reduced by CNTF* in vivo *[211]. CNTF was shown to potentiate peripheral nerve regeneration after transection and juxtaposition of the sciatic nerve in rats by promotion of a higher number of elongating axons into the distal stump [212]. Sprouting can also be induced when exogenous CNTF is applied to a partial denervated muscle in homozygous CTNF^−/−^ knockout mice, which are otherwise not capable of neural sprouting [213, 214]. BDNF and CTNF used in conjunction with a collagen tube in the repair of sciatic nerve defects in rats yielded more favorable functional recovery than either the collagen tube or BDNF and CTNF alone [215].

### 8.8. NT-3 and NT-4/5

The roles of NT-3 and NT-4/5 in promoting survival of injured motor neurons are not entirely explored [216]. However, evidence suggests that NT-3 and NT-4/5 might be as effective as BDNF in promoting the survival of injured motor neurons, although there are several studies that showed lesser effects on the survival of axotomized motor neurons compared to BDNF [217–219]. Moreover, Koliatsos et al. showed that there was no influence of NT-3 on the survival of motor neurons [193].

### 8.9. NRG-1

There are six known main isoforms of NRG-1 (with greater than 30 subforms): I, II, and III are restricted to vertebrates, isoform IV is restricted to mammals, and isoforms V and VI are restricted to primates [220]. Because of their importance in nerve regeneration, NRG-1 isoforms I, II, and III gained increasing scientific interest over the last few years [221]. The promyelinating activity of NRG-1 depends on the isoform and concentration. Promyelinating activity is promoted, for example, by a broad dose range of NRG-1 isoform III but only through very low concentrations of isoform II [222]. Stassart et al. showed a peak production of NRG-1 isoforms I and II after peripheral nerve injury [223]. Data from current studies indicate that in large lesions Schwann cells are not capable of producing the required amounts of NRG-1 and soluble NRG-1 isoforms I and II to improve nerve repair [220, 224]. Nicolino et al. demonstrated that Schwann cell proliferation and migration are accompanied by NRG-1 upregulation [225]. Several studies indicated an improvement in remyelination through the usage of different soluble NRG-1 isoforms [222, 223, 226, 227]. Gambarotta et al. recommended tissue engineering nerve conduits with a high concentration of NRG-1 for Schwann cell dedifferentiation, followed by a late, less concentrated NRG-1 release to support remyelination [220].

## 9. Low Frequency Electrical Stimulation for Nerve Regeneration/Electrically Conducting Polymer 

The role of electrical stimulation as a therapeutic intervention in patients with nerve injury, for example, the facial nerve, has been controversially discussed among experts [228–238]. A recent study conducted by Kim and Choi showed a promising effect of subthreshold continuous electrical stimulation at 20 Hz, through a surface electrode on the facial function of 60 patients with Bell's palsy [232]. Based on a myriad of studies, several cell types including cardiomyocytes, neurons, and osteoblasts respond to electrical signals by improving their functional outcomes.

Improved functional outcome after application of electrical signals was demonstrated* in vivo *and* in vitro* in several cell types including neurons, cardiomyocytes, and osteoblasts. Peripheral nerve regeneration has been demonstrated in studies using external electrical stimulation, piezoelectric guidance channels, injected electrets, polymer electret guidance channels, and electrically conductive polymers [239–243]. A variety of conducting polymers are available, including polyaniline (PANI) and polypyrrole (PPy) which have attractive properties such as ease of synthesis, tunable conductivity, environmental stability, and biocompatibility [244, 245]. Furthermore, these polymers deliver electrical cues to target sites and simultaneously provide physical support for cell growth [246]. In an* in vitro* study Lee et al. produced and tested an electrospun PLGA scaffold that was coated with PPy and NGF molecules (NGF-PPyPLGA). Applying electrical potential (10 mV/cm) through the conducting fibers resulted in promising improvement of neurite development and neurite length of the NGF-immobilized fibers compared to unstimulated cells [244].

## 10. Towards Clinical Practice in Craniomaxillofacial and Facial Plastic Surgery

Only a minor part of the above-mentioned ongoing research strategies and approaches concerning artificial nerve guidance conduits has yet been applied to preclinical experimental studies or clinical studies in the craniomaxillofacial area and particularly the facial nerve (Tables 4 and 5).

Preclinical* in vivo* experiments have been conducted on the nerve regeneration-promoting effect in facial nerve injuries. 7 mm nerve facial defects in rats have been reconstructed with nonwoven polylactic acid tubes. In this work, the PLA nonwoven fabric tube, composed of randomly connected PLA fibers, showed better histological outcome compared to a silicon tube control [247]. Also stem cell studies on facial nerve reconstruction have found their way into experimental* in vivo* research. In a study by Shi et al., rats underwent facial nerve transection and subsequent reconstruction with polyglycolic/polyglycolic acid (PGA) nerve guidance conduits filled with neural stem cells (NSC) overexpressing glia-derived neurotrophic factor. This combination yielded a significantly greater nerve action potential amplitude, axonal area, and axonal number compared to empty control group conduits or even to NSC conduits without overexpressing factors [172]. Bone marrow stem cells (BMSCs) and Schwann-like cells were used in a further study also in combination with PGA tubes to evaluate facial nerve regeneration [173]. Here they concluded that regeneration of the facial nerve was improved by using BMSC in PGA tubes in rats, although Schwann-like cells still yielded superior results. Guo and Dong observed comparable results to an autograft in 10 mm facial nerve defects of rabbits when using a chitosan conduit, which contains neural stem cells [38]. Neurotrophic factors like NGF were implemented in the form of microspheres within chitosan conduits for sustained release in order to regenerate 10 mm facial nerve defects in rabbits. The study yielded significantly improved facial nerve defect repair compared to chitosan conduits combined with regular nerve growth factor [35]. Biomaterials like silk fibroin-chitosan conduits (SFCS) have also been studied successfully on the facial nerve. Silk fibroin scaffolds used in nerve regeneration of rabbits with a 10 mm facial nerve defect suggest a possible substitute for the conventional autograft technique [37]. Cui et al. used a functional collagen nerve conduit incorporated with neurocytokines to bridge a 35 mm long facial nerve gap in minipig models in order to evaluate facial nerve regeneration over longer distances than in rodent models [32]. First translations from bench to bedside have been published in preliminary clinical studies. A case report from Inada et al. showed favorable results of bridging facial nerve defects in two patients undergoing nerve reconstruction with a polyglycolic acid (PGA) tube filled with collagen [33]. Case studies on facial nerve defects reconstructed with FDA approved ANCs are summarized in Table 4. The outcome of these case studies reveals promising preliminary results as in most cases functional recovery could be attained by the use of artificial nerve guidance conduits.

## 11. Summary and Future Perspectives

Well-known limitations of autografts include the need of a second surgery site, donor site morbidity, limited length of grafts, and mismatch of nerve size. Only 40 to 50% of the patients treated with an autologous nerve graft regain an acceptable level of function [14]. Therefore, research involving neural tissue engineering has evolved tremendously over the last decade. Through increasing knowledge of mechanisms underlying the complex process of nerve regeneration, developments in nanofabrication, polymers, gene and growth factor delivery, and stem cell technologies enable the design of new generations of conduits that increasingly resemble the features for human neural regeneration [129]. Electrospinning and biofactor mobilization are techniques that improve biocompatibility and control inner conduit bioactivity. Lumen fillers that are seeded with stem cells or Schwann cells provide growth guidance and growth factor delivery. Furthermore, the development of conduits with electroconductive material, and conductive polymers may improve and fasten the nerve regeneration process. Major advances in neurobiology and the material sciences, for example, the 3D printing of material or the possibility of expressing growth and differentiation factors on silk fibers by means of bioengineered silk worms (*Bombyx mori*) for the production of conduit material, provide an exciting prognosis for the future. Despite the major progress in the development of artificial nerve guidance conduits, to date no specificity of reinnervation of appropriate target structures has been accomplished. Thus, inappropriate reinnervation and cocontractures remain a problem for future repair strategies. Regarding the craniomaxillofacial aspect, biodegradable nerve tubes as alloplastic alternative have not found their way towards clinical practice yet, as to date only preclinical results and case reports have been published [248, 249] (Tables 4 and 5). In the background of its epidemiological relevance and severe impact on the quality of life aspect, studies on ANCs reconstructing the facial nerve should be broadened and given more consideration in future experimental works. Research and further multicenter trials on a large scale are needed in conjunction with an understanding of spatiotemporal requirements in neural regeneration to replace the gold standard of an autologous nerve graft and to facilitate the translation of artificial nerve guidance conduits from bench to bedside in craniomaxillofacial surgery.

## Figures and Tables

**Table 1:** Sunderland classification.

Sunderland class	Injury	Recovery
I	*Neuropraxia*: localized and reversible conduction blockade	Complete
II	*Axonotmesis*: axonal disruption	Complete
III	*Endoneurium*: axonal and endoneurial sheath disruption	Wallerian degeneration, incomplete
IV	*Perineurium*: axonal, endoneurial sheath and perineurial sheath	Wallerian degeneration, incomplete
V	*Neurotmesis*: axonal, endoneurial sheath, perineurial sheath, and epineurial sheath	Wallerian degeneration, incomplete

**Table 2:** The table is presented with permission of Jowett and Hadlock [29].

Term	Definition
Facial palsy (FP)	Term encompassing entire spectrum of facial movement disorders including facial paralysis, flaccid facial palsy, and nonflaccid facial palsy
Facial paralysis	Complete absence of facial movement and tone
Flaccid facial palsy (FFP)	Absence or weakness of facial movement and tone, without synkinesis or hyperactivity
Nonflaccid facial palsy (NFFP)	A postparetic state whereby aberrant nerve regeneration has occurred, consisting of varying degrees of zonal synkinesis and hypoactivity and hyperactivity
Facial synkinesis	Involuntary and abnormal facial muscle activation accompanying volitional or spontaneous expression

**Table 3:** FDA approved nerve guidance conduits.

Product	Material	Structure	Degradation time	Company	FDA-approval
NeuroTube	Polyglycolic acid	Absorbable woven mesh tube	3 mo	Synovis Micro Companies	1999
NeuraGen	Type I collagen	Semipermeable, fibrillar	3-4 yrs	Integra LifeSciences Co., Plainsboro, NJ, USA	2001
NeuroFlex	Type I collagen	Semipermeable, flexible, tubular	4–8 mo	Collagen Matrix, Inc., Franklin	2001
NeuroMatrix	Type I collagen	Semipermeable, flexible, tubular	4–8 mo	Collagen Matrix, Inc.	2001
NeuraWrap	Type I collagen	Semipermeable, longitudinal slit in wall	36–48 mo	Integra LifeSciences Co.	2004
NeuroMend	Type I collagen	Semipermeable wrap, unrolls and self-curls	4–8 mo	Collagen Matrix, Inc.	2006
Neurolac	Poly-DL-lactide-caprolactone	Synthetic and transparent, tubular	16 mo	Polyganics BV, Groningen, Netherlands	2003
SaluTunnel	Polyvinyl alcohol	Nonbiodegradable	No degradation	Salumedica LLC, Atlanta, GA, USA	2010
Avance	Processed human nerve allograft			AxoGen, Inc., Alachua, FL	2010
AxoGuard	Extracellular matrix derived from porcine small intestine submucosa	Absorbable semipermeable	No data	AxoGen, Inc., Alachua, FL	2013

**Table 4:** Preclinical and experimental studies on facial nerve reconstruction by artificial nerve guidance conduits.

Study	Year	Conduit material	Cells/factors	Species	Defect size or technique	Regrowth time span (weeks)	Outcome
Cui et al. [32]	2014	Collagen	Neurocytokines CNTF and bFGF	Minipig	35 mm	24 weeks	(i) Favorable mechanical properties (ii) May promote facial nerve regeneration effectively

Inada et al. [33]	2007	Polyglycolic acid collagen blend	None	Human	n/a	24 weeks	(i) Functional and morphological regeneration

Matsumine et al. [34]	2014	Polylactic acid, nonwoven	None	Rat	7 mm	13	(i) Comparable ability of autografts to induce peripheral nerve regeneration

Liu et al. [35]	2013	Chitosan	Nerve growth factor presented in microspheres	Rabbit	10 mm	13	(i) Sustained release of nerve growth factor can significantly improve facial nerve defect repair

Shi et al. [36]	2012	Polylactic-co-glycolic acid	Neural stem cells (NSC)	Rat	Facial nerve transection	12	(i) Nerve action potential amplitude and axonal area were significantly greater in the NSC compared to an empty control group

Tan et al. [37]	2009	Silk fibroin-chitosan blend	None	Rabbit	10 mm	8	(i) Successful regeneration of the facial nerve

Guo and Dong [38]	2009	Chitosan	Neural stem cells (NSC)	Rabbits	10 mm	12	(i) Comparable results to an autograft in 10 mm facial nerve defects

**Table 5:** Clinical studies on facial nerve reconstruction with FDA approved nerve guidance conduits.

Study	Year	Product	Type of research	Species	Defect/pathology	Case count	Functional recovery attained
Navissano et al. [39]	2005	NeuroTube	Case report	Human	1–3 cm	7	Yes (71% of the cases)
Dwivedi et al. [40]	2006	NeuraGen	Case report	Human	Hypoglossal-facial anastomosis	1	Yes
Gunn et al. [41]	2010	Avance	Case report	Human	Paraganglioma	1	Yes
Brant et al. [42]	2016	Avance/AxoGuard	Technical report	Human	Facial nerve schwannoma	1	n/a
